# Supplementary material for: Large language models for disease diagnosis: a scoping review
Source: NPJ Artif Intell. 2025 Jun 9;1(1):9. doi: 10.1038/s44387-025-00011-z (PMC12216946; doi:10.1038/s44387-025-00011-z)
Supplement: Supplementary file 1 — Supplementary information [file 44387_2025_11_MOESM1_ESM.pdf]

Supplementary Data 1: Search Strategy (list of queries used to search databases)

Index	Search terms
1	“large language model” AND (“clinical note” OR “clinical text” OR “health record”) AND (“diagnosis” OR “clinical decision making”)
2	“large language model” AND “medical conversation” AND (“diagnosis” OR “clinical decision making”)
3	“large language model” AND “medical dialogue” AND (“diagnosis” OR “clinical decision making”)
4	“large language model” AND “medical image” AND (“diagnosis” OR “clinical decision making”)
5	“large language model” AND (“CT” OR “X-ray” OR “MRI” OR “Ultrasound” OR “Fluoroscopy” OR “Endoscopy”) AND (“diagnosis” OR “clinical decision making”)
6	“large language model” AND “video” AND (“disease diagnosis” OR “clinical decision making”)
7	“large language model” AND “report generation” AND (“diagnosis” OR “clinical decision making”)
8	“large language model” AND “Multi-modal” AND (“diagnosis” OR “clinical decision making”)
9	“large language model” AND “visual question answering” AND (“diagnosis” OR “clinical decision making”)
10	“large language model” AND (“mental health” OR “psychology”) AND (“disease diagnosis” OR “clinical decision making”)
11	“large language model” AND “gene” AND (“diagnosis” OR “clinical decision making”)
12	“large language model” AND (“sensor” OR “time series”) AND (“disease diagnosis” OR “clinical decision making”)
13	“large language model” AND “tabular data” AND (“disease diagnosis” OR “clinical decision making”)
14	“large language model” AND (“speech” OR “audio”) AND (“disease diagnosis” OR “clinical decision making”)
15	“large language model” AND “disease” AND “risk prediction”

Supplementary Table 1: Data Extraction Form

Concept	Definition
**Basic information**	
ID	Unique ID assigned to each paper.
Paper title	The title of the included paper.
Venue	The venue of the paper, including preprint platforms, e.g., arXiv or medRxiv.
Published time	The year and month in which the study was published.
Corresponding region	The continent of the corresponding author.
**Data information**	
Data source (region)	The sources (continents) of the exploited datasets. If the datasets are from multiple sources, we listed all of them.
Disease type	The name of the investigated diseases in the study.
Clinical specialty	The disease-associated clinical specialty of the study. If multiple diseases were involved, we wrote down up to three closely related specialties.
Clinical data type	The type of the leveraged (clinical) data, such as clinical note, CT, MRI, patients' dialogues, and clinical guidelines.
Data modality	The data modality of the leveraged data, including text, image, video, audio, time series, graph, tabular data, omics, and various of multi-modal cases.
Data size	The total size of the leveraged data.
Dataset name	The name of the leveraged dataset.
Data privacy status	Whether the applied dataset was public available. If the study involved private data and public data, we wrote down "Both".
**Model information**	
Base LLM type	The leveraged base LLM in the study, e.g., GPT-4 or T5.
Parameter size	The parameter number of the LLM in this study. If the information is unavailable, we wrote it as "NA".
LLM technique	The leveraged LLM techniques in the study, e.g., prompt.
**Evaluation**	
Evaluation schema	The evaluation strategy used in the study, i.e., human evaluation, automatic evaluation, or LLM evaluation.
Evaluation metric	The specific evaluation metrics in the study.

Supplementary Data 2: Scope of Disease Diagnosis

Automatic disease diagnosis is usually defined as receiving clinical data, such as patient symptoms, medical history, and diagnostic tests, as input and using computational models to identify which disease explains the presented symptoms and signs. In this review, the scope of disease diagnosis was not confined to the studies that directly output the diagnosis but also encompassed the studies that output probability values of certain diseases, e.g., the probability of suffering from depression. The main reason is that early diagnosis is essential in clinical scenarios. Considering that many diseases do not present obvious symptoms or signals in the early stages, providing probability values is also of great significance and should be taken into scope. Notably, we exclusively removed all studies related to disease prognosis from our review paper since they highlight predicting the likely outcome or course of a disease, including the patient’s chance of recovery, survival, or the risk of mortality.

We listed some representative diagnostic tasks below.

- Disease diagnosis: The studies that receive clinical data as input and generate the most possible diagnosis.
- Differential diagnosis: The studies that receive clinical data as input and generate a set of most likely diagnoses that are associated with similar conditions.
- Conversational diagnosis: The studies that interactively collect patients’ symptom information through medical conversation and output the diagnoses.
- Diagnostic report generation: The studies that utilize medical images to generate diagnoses and clinical reports. Notably, only the studies that could generate diagnoses were included.
- Risk prediction: The studies that receive clinical data as input and predict potential risk probabilities, such as the probability of suffering from heart failure. Notably, we excluded the studies involving disease prognosis, such as survival analysis.
- Mental health disorder detection: The studies that receive clinical data as input and detect potential illnesses in mental health, such as depression detection or cognitive distortion detection.
- Medical image classification: The studies that receive medical images as input and output the corresponding disease types.
